# Universality of size-energy ratio in four-body systems

**DOI:** 10.1038/s41598-019-42312-9

**Published:** 2019-04-18

**Authors:** Petar Stipanović, Leandra Vranješ Markić, Andrii Gudyma, Jordi Boronat

**Affiliations:** 1 University of Split, Faculty of Science, R. Boškovića 33, HR-21000 Split, Croatia; 2 Departament de Física, Universitat Politècnica de Catalunya, Campus Nord B4-B5, E-08034 Barcelona, Spain

**Keywords:** Structure of solids and liquids, Scaling laws, Quantum physics, Atomic and molecular physics

## Abstract

Universal relationship of scaled size and scaled energy, which was previously established for two- and three-body systems in their ground state, is examined for four-body systems, using Quantum Monte Carlo simulations. We study in detail the halo region, in which systems are extremely weakly bound. Strengthening the interparticle interaction we extend the exploration all the way to classical systems. Universal size-energy law is found for homogeneous tetramers in the case of interaction potentials decaying predominantly as *r*^−6^. In the case of mixed tetramers, we also show under which conditions the universal line can approximately describe the size-energy ratio. The universal law can be used to extract ground-state energy from experimentally measurable structural characteristics, as well as for evaluation of theoretical interaction models.

## Introduction

Universality connects phenomena in various fields of physics and enables to discern properties in a hardly accessible field by studying some easier model problems. In few-body physics, universality is often observed in weakly-bound systems of particles with average interparticle distances larger than the range of their interactions. It is manifested as the independence of some system properties on the exact shape of the interaction potential and on the length scale.

One of the most known phenomenon in few-body physics is the Efimov prediction^1^ on the geometric series of three-body bound-state levels in the unitary limit, i.e., when a two-body state is exactly at the dissociation threshold. The observation of this singular prediction remained an elusive goal for decades. For a long time, dineutron halo nuclei^2–4^ were considered as the most promising Efimov candidates. Although naturally existing nuclei are not close to a resonance to clearly show the Efimov effect, their study encouraged the search of universality in atomic and molecular physics, and subsequently in condensed matter, all the way up to high-energy physics^5,6^. The first signature of the Efimov effect appeared in an ultracold gas of Cesium atoms^7^ due to the high tunability of their interactions. The most promising stable Efimov candidate, the atomic cluster ^4^He_3_, which is weakly-bound under natural conditions, was recently observed by using Coulomb explosion imaging^8^. The discovery of the Efimov effect^1^ naturally opened the question whether the same effect could exist in *N*-body systems, but it was concluded rather soon^9^ that there is no true Efimov effect for four or more identical bosons. Despite of that the prediction of a variety of universal bound states linked to the Efimov trimer motivated new research that was extended^5,10–12^ to non-identical particles and (*N* > 3)-body systems.

The search of the Efimov effect in nuclei opened discussions about the ground-state universality of two- and three-body halo states^2–4^, i.e., of few-body systems which prefer to be in classically forbidden regions of space. Atomic clusters, which are small aggregates of atoms^13,14^ whose interactions are well known, played a significant role in elucidating universal ground-state size-energy ratios in weakly-bound dimers and trimers^15^. In fact, some structural properties of atomic clusters are becoming experimentally feasible. Particle distribution functions in dimers, trimers, and tetramers of argon and neon were recently measured by Coulomb explosion imaging^16^, as well as in weakly-bound helium trimers^8,17,18^ and the dimer ^4^He_2_^19^. Extensions of the imaging approach applied in the case of helium trimers was qualified as feasible for the four-body sector^8^. Recently, four-body halo and quasi-halo states were predicted to exist in helium tetramers^20^ and in tetramers obtained by swapping one He atom by an alkali one^21^. Moreover, a thorough analysis for a large set of pure and mixed weakly-bound atomic dimers and trimers showed that universal size-energy scaling extends even below the halo area^15^, in the so called quasi-halo region. Motivated by that, in this work we extend our ground-state universality research to four-body systems including the regime of strongly interacting systems of identical and non-identical particles.

Universal phenomena are usually compared in different fields of physics, where characteristic scales differ in many orders of magnitude, and thus one introduces dimensionless quantities. Dimensionless scaling of two-body size follows straightforwardly from the halo definition^3^ using the mean square radius, and dividing it by the outer classical turning point, 〈*r*^2^〉*R*^−2^ ≥ 2. This condition assures that the probability of occupying the classically forbidden region of space is larger than 50%. The three body-systems are obviously more complex. Some pairs can be self-bound, whereas others not; some pairs can be in a classically forbidden region, while others in a classically allowed region. In this line, the size of a three-body halo state was quantified^22^ as the mean value of the square hyperradius *ρ*^2^, i.e. the mass-weighted radial momenta of all particles with respect to the center of mass **R**_CM_. This definition of the size for the three-body system^22^, can be generalized for a *N*-body system as

1 $${\rho }_{r}^{2}=\sum _{i}^{N}\frac{{m}_{i}}{m}\langle {({{\bf{r}}}_{i}-{{\bf{R}}}_{{\rm{C}}{\rm{M}}})}^{2}\rangle =\frac{1}{Mm}\sum _{i < k}^{N}{m}_{i}{m}_{k}\langle {r}_{ik}^{2}\rangle ,$$

where the total mass *M* and masses of particular particles *m*_*i*_ are given in an arbitrary mass unit *m*, which is included in the definition (1) for convenience. The subscript *r* in ${\rho }_{r}^{2}$ stands for a measure of the size of the system using the mean square distances $\langle {r}_{ik}^{2}\rangle$ between particles *i* and *k*. This size is then scaled as ${Y}_{\rho }={\rho }_{r}^{2}{\rho }_{R}^{-2}$, where the subscript *R* denotes the use of squared characteristic lengths ${R}_{ik}^{2}$ in Eq. (1) instead of $\langle {r}_{ik}^{2}\rangle$. Our goal is to show how the scaled size of the four-body systems *Y*_*ρ*_ depends on the dimensionless energy ${X}_{E}=mB{\rho }_{R}^{2}{\hslash }^{-2}$, where *B* = |*E*| is the absolute value of the energy.

In previous research^15^, we showed that the width *R* = *R*_e_ of a square well potential, which has the same integral properties as the realistic potential *V*(*r*), is convenient for the scaling of two- and three-body halo states. The condition for having a halo state is then generalized to *Y*_*ρ*_(*R* = *R*_e_) ≥ 2. The scaling length *R*_e_ can be calculated in the regime where the atom-atom scattering is dominated by *s*-wave phase shifts and is thus not applicable to strongly bound systems. In this line, we chose the van der Waals length *R* = *R*_6_ = 2*l*_vdW_^5,23^ as additional scaling length, which can be easily calculated for arbitrarily strong pair potential, as is described in the next Section. The choice was motivated by the simple relationship between the effective range, scattering length and *R*_6_ for the pure atom-atom van der Waals potential −*C*_6_*r*^−6^ in the ultracold regime; and by the van der Waals universality for three cold atoms near Feshbach resonances^5,6,24–26^. In fact, Jia Wang *et al*.^25^ found that the three-body potential, regardless of the existence of a repulsive core in the two-body potential, exhibits a steep barrier which prevents the three particles from getting close together, thus preventing configurations with small hyperradii, *ρ* > *R*_6_. In addition, it was shown for homogeneous three-body systems, whose long-range pair interactions are dominated by −*C*_6_*r*^−6^, that the ground state trimer dissociation scattering length ${a}_{-}^{\mathrm{(0)}}$ is universally proportional to *l*_vdW_. For broad atomic resonances, the choice^5^

2 $${a}_{-}^{\mathrm{(0)}}=-\,9{l}_{{\rm{vdW}}}\pm 20 \% $$

covers possible discrepancies between the current theoretical and somewhat lower experimental results^5,6,24–26^. Higher-order contributions to the van der Waals tail are believed to cause those deviations. For that reason, in this work we do not only test different scaling lengths, but also different interatomic potential models.

In the quantum halo regime of nuclei, it was shown^22,27,28^ that three-body systems are the more compact the lower is the number *N*_2_ of self-bound sub-dimers (for the same binding energy). Namely, tuning the scattering lengths from large and positive to large and negative, pair interactions become less attractive and, to keep the same binding, the three-body system shrinks. Relevant universal features in three-body systems were recently confirmed using a large set of atomic clusters^15,29^. The scaled sizes increased following the sequence^22,27,28^ *N*_2_ = 0, 1, 2, 3, where respective trimers were named Borromean, tango, samba, and all-bound. Since there is more variability in four-body systems, they are more challenging than three- and two-body systems. Therefore, the existence of tetramer size ordering, in a similar form to the previously discussed sequence for trimers, can not be stated *a priori*. In the present work, we classify the four-body systems according to the number of self-bound sub-dimers and -trimers which enables us to investigate if and under what circumstances sequences of tetramer sizes emerge.

## Results

**Figure 1:**
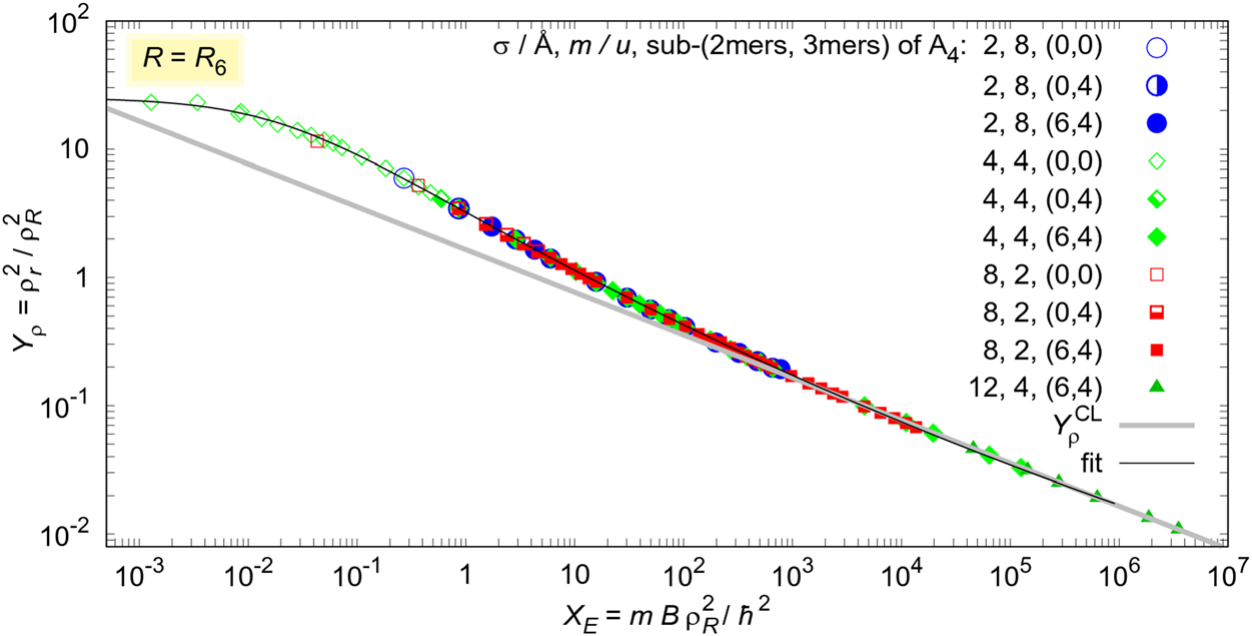
Scaled size-energy fit for various homogeneous quantum four-body systems A_4_. Interactions are modeled by the Lennard-Jones 6–12 pair potential. Tetramers are classified according to the number of self-bound subsystems of dimers and trimers. Different systems types, mass of particle *m* and size of repulsion core *σ* are distinguished by different symbol types. The van der Waals length was used for scaling, *R* = *R*_6_. For comparison, we report the classical result, with ${Y}_{\rho }^{{\rm{CL}}}$ given by Eq. (5).

We discuss first homogeneous four-body quantum systems, where the term homogeneous stands for clusters of four identical particles. Their ground-state properties are obtained by solving *exactly* the imaginary-time Schrödinger equation using quantum Monte Carlo methods. Different masses of particles are explored but, for simplicity, we use only multiples of the atomic unit of mass *u*. Atom-atom interactions are modeled by the Lennard-Jones 6–12 pair potential *V*(*r*) = 4*ε*[(*σ*/*r*)^12^ − (*σ*/*r*)^6^], which is extremely repulsive and positive for small *r* < *σ*, reaches its minimum −*ε* at ${r}_{{\rm{m}}}=6\sqrt{2}\sigma$, and further on becomes attractive and decays to zero following the van der Waals tail −*C*_6_*r*^−6^. For particles of masses 8, 4, 2, 4 *u*, we used repulsive cores *σ* = 2, 4, 8, 12 Å, respectively. For the four *σ* values, in increasing order, we used potential depths in the ranges 7.5–45, 3.55–330, 1.8–45, and 20–300 K. We explored a wide range of binding strengths, with ground-state energies ranging from 0.2 mK to 1.65 kK. The obtained energies *X*_*E*_ and sizes *Y*_*ρ*_ are shown in Fig. 1 as points. As reduced unit length we used the van der Waals length *R* = *R*_6_, i.e., the solution of the equation *ℏ*^2^/(2 *μR*^2^) = *C*_6_*R*^−6^, where *μ* is the reduced mass of a given pair. Different combinations of masses and core sizes are distinguished by different symbol shapes (circles, diamonds, squares, and triangles). The condition for a quantum halo when using the *R*_6_ as the scaling length is *Y*_*ρ*_ > 5.

For a given tetramer ABCD, one can have four different sub-trimers: ABC, ABD, ACD, and BCD, which are or are not self-bound, depending on the interparticle interaction and masses. Also, there are six possible pairs, which are or are not self-bound. In the homogeneous system all the pairs are equivalent, so we can have in total 0 or 6 self-bound sub-dimers, and 0 or 4 self-bound sub-trimers. We denote the tetramer type as (*N*_2_, *N*_3_), where *N*_2_ and *N*_3_ are the numbers of self-bound sub-dimers and sub-trimers, respectively. Because of the pair equivalence, the homogeneous systems can form only (0, 0), (0, 4) and (6, 4) types, which are reported in Fig. 1 by different symbol filling: empty, half-full, and full, respectively.

As one can see, all homogeneous quantum tetramers in Fig. 1 follow the same law, regardless of the mass, interaction potential, and number of self-bound sub-systems. Below a maximum energy *X*_*E*_ < 10^6^, our quantum Monte Carlo results are well reproduced by the function

3 $$Y(X)=\exp \{{Y}_{0}+{({X}_{0}+\xi {X}^{k})}^{n}\}\mathrm{.}$$

The parameters of the best fit are given in the first row of Table 1 (*R* = *R*_6_).

**Table 1:** Fitting parameters.

*R*	*Y*_0_	*X*_0_	*ξ*	*k*	*n*
*R*_6_	−12.626	6.78E-29	1.73E-27	0.82	−0.04261
*R*_e_	−0.248	6.86E-2	1.67E-1	0.62	−0.46561
*R*_o_	−2.598	9.76E-29	2.48E-27	0.82	−0.04283
*R*_io_	−2.768	4.73E-27	1.10E-25	0.76	−0.04604

**Figure 2:**
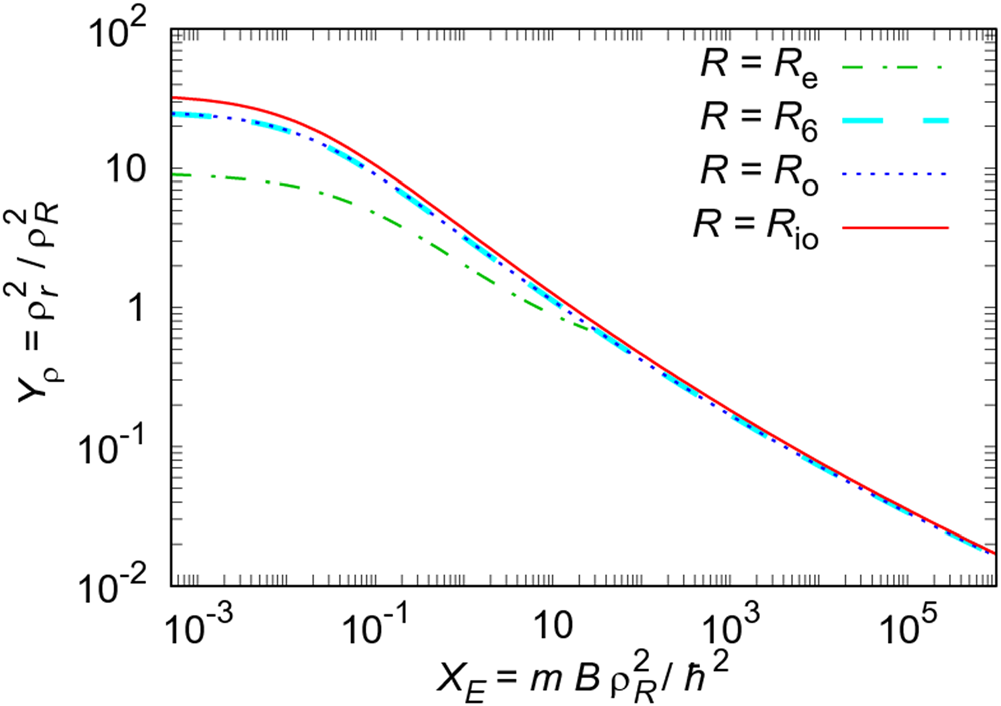
Comparison of scaled size-energy fits for homogeneous tetramers. Different scaling lengths were used: the width of the equivalent square well *R*_e_, the van der Waals length *R*_6_, the lengths *R*_i,o_ obtained generalizing the *R*_6_ definition to the whole potential form, ${\rho }_{{R}_{{\rm{i}}{\rm{o}}}}^{2}={\rho }_{{R}_{{\rm{o}}}}^{2}-{\rho }_{{R}_{{\rm{i}}}}^{2}$.

Equation (3) was fitted to the data from Fig. 1, after rescaling points using different lengths *R*. The functions are compared in Fig. 2.

For the sake of comparison, **classical four-body systems** were also analysed at zero temperature, where no thermal motion is possible. In this case, particles occupy the minima of the pair potential *V*(*r*) = *ε*[(*r*_m_/*r*)^*k*^ − 2(*r*_m_/*r*)^*n*^], *k* > *n*. The minimal energy configuration corresponds to a tetrahedron, whose vertex corners are separated by a distance *r*_m_. For the Lennard-Jones 6–12 potential ${r}_{{\rm{m}}}=6\sqrt{2}\sigma$. Therefore, the binding energy is *B* = 6*ε* and the size ${\rho }_{r}^{2}=0.5{r}_{{\rm{m}}}^{2}$. By introducing the generalized quantum scaling length

4 $${R}_{n}={(0.5M\varepsilon {r}_{{\rm{m}}}^{n}{\hslash }^{-2})}^{\frac{1}{n-2}}$$

one gets a simple relation between scaled size and energy for the classical systems,

5 $${Y}_{\rho }^{{\rm{CL}}}={(\mathrm{4.5/}{X}_{E})}^{\frac{2}{n}}.$$

Equation (5) for *n* = 6 is compared with the quantum Monte Carlo results in Fig. 1. The classical line (5) smoothly continues after the quantum fit, below *Y*_*ρ*_ ≈ 0.02. Overlap between quantum and classical results is clearly visible for *Y*_*ρ*_ < 0.2, while for higher scaled sizes separation occurs. As the scaled energy decreases, the probability of occupying the classically forbidden region of the space increases. It becomes significant in the quasi-halo region 0.2 < *Y*_*ρ*_ < 5, and dominates in the halo region *Y*_*ρ*_(*R* = *R*_6_) > 5. ${Y}_{\rho }^{{\rm{CL}}}$ manifestly underestimates the size in the quasi-halo region and the difference with the quantum behavior continuously increases in the halo region, as the scaled energy *X*_*E*_ decreases towards 10^−2^. The size of the quantum clusters converges to *Y*_*ρ*_ ≈ 25 at the binding threshold. Contrarily, the classical scaled size diverges and therefore intersects the quantum law.

Although the lines overlap when *Y*_*ρ*_ < 0.2, the classical and quantum calculations do not predict the same point on the line for a particular system. Classical calculations always underestimate the size, and overestimate the energy because they do not include any kinetic energy contribution. For instance, solving the Schrödinger equation for a four-body system of total mass *M* = 16*u*, with pair interactions *V*(*r*) = 80 K[(4 Å/*r*)^12^ − (4 Å/*r*)^6^], we get *B* = 32.38 K and 〈*r*^2^〉 = 32 Å^2^, i.e., *X*_*E*_ = 660 and *Y*_*ρ*_ = 0.20. These values are significantly different from the classical predictions: 37% lower size and four times higher energy. Of course, the difference decreases when binding increases. The classical approximation for the bottom point in Fig. 1 gives only a 3% lower size, and a 9% larger binding energy; the correction agrees with the ratio of kinetic and potential energies. On the other hand, if we consider a quantum halo system, which has *Y*_*ρ*_ = 7, the classical calculation predicts 96% lower size, and almost 1100 times higher energy.

The scattering lengths depend on the mass of the particles. This basic feature and the variety of self-bound sub-dimers and -trimers make tetramers much more complex than the trimers^22,27,28^. The top part of the line in Fig. 1 is occupied only by (0, 0) systems, both (0, 4) and (6, 4) types appear in the middle, and only (6, 4) are found at the end of the line which corresponds to strongly bound systems. If one fixes the mass, the ordering *Y*_*ρ*_(*N*_2_ = 0, *N*_3_ = 0) > *Y*_*ρ*_(0, 4) > *Y*_*ρ*_(6, 4) is noticeable. Furthermore, our results in Fig. 1 predict in general *Y*_*ρ*_(0, 0) > *Y*_*ρ*_(0, 4) and *Y*_*ρ*_(0, 0) > *Y*_*ρ*_(6, 4), without fixing any quantity.

The strongest bound (0, 0) tetramers in Fig. 1, represented by the lowest empty symbols, are very close to the three-body dissociation threshold and thus can be used to verify the Eq. (2). For instance, we considered tetramers (4*u*)_4_ and (2*u*)_4_ of identical particles with mass 4*u* and 2*u* in atomic units, respectively (*u* = 1.66053904 × 10^−27^ kg). The scattering lengths for the pairs 4*u* − 4*u* and 2*u* − 2*u*, with interacting parameters *σ* = 4, 8 Å and *ε* = 3.85, 1.9 K, respectively, are −34 Å and −63 Å, which are in agreement with the evaluation using Eq. (2), −38(8) Å and −76(15) Å.

Different scaling lengths were also tested. Parameters obtained using the homogeneous tetramer data scaled with different scaling lengths are given in Table 1, while the comparison of the different scalings is shown in Fig. 2. The value *R*_e_ obtained from the scattering-equivalent square well, which resulted in universal dimer and trimer size-energy laws^15^, is valid only in the regime of low-energy scattering. Thus, it was used only for *X*_*E*_ < 20. In order to examine higher-order long-range potential corrections, we generalized the *R*_6_ definition using the full potential form *V*(*R*) instead of the asymptotic form −*C*_6_*R*^−6^. For every interacting pair, we found the inner and outer solutions of *ℏ*^2^/(2*μR*^2^) = *V*(*R*), *R*_i_ < *R*_o_. The sizes and energies, rescaled by the generalized hyper-radii ${\rho }_{{R}_{{\rm{o}}}}^{2}$ and ${\rho }_{{R}_{{\rm{i}}{\rm{o}}}}^{2}={\rho }_{{R}_{{\rm{o}}}}^{2}-{\rho }_{{R}_{{\rm{i}}}}^{2}$, are hardly distinguishable for strong binding. As binding becomes weaker, i.e., when *X*_*E*_ becomes lower, the *R*_io_ law goes slightly above *R*_6_ line, while *R*_o_ line is within the errorbar above the *R*_6_ line. The scaled sizes approach constant values in the limit of weak binding for all *R*. This is in agreement with the study of four-body Brunnian systems^30^ and with the recent analysis on the validity limits of Efimov physics^12^. Using the stochastic variational method, both studies concluded that the radius of few-body clusters approaches a constant value for very weak binding. Those works used a different scaling length *R* = *b*, which is applicable only in the case of the pair potential *V*(*r*) = *V*_0_ exp(−*r*^2^/*b*^2^), so scaled sizes are different, as we also noticed here for different *R* values.

**Figure 3:**
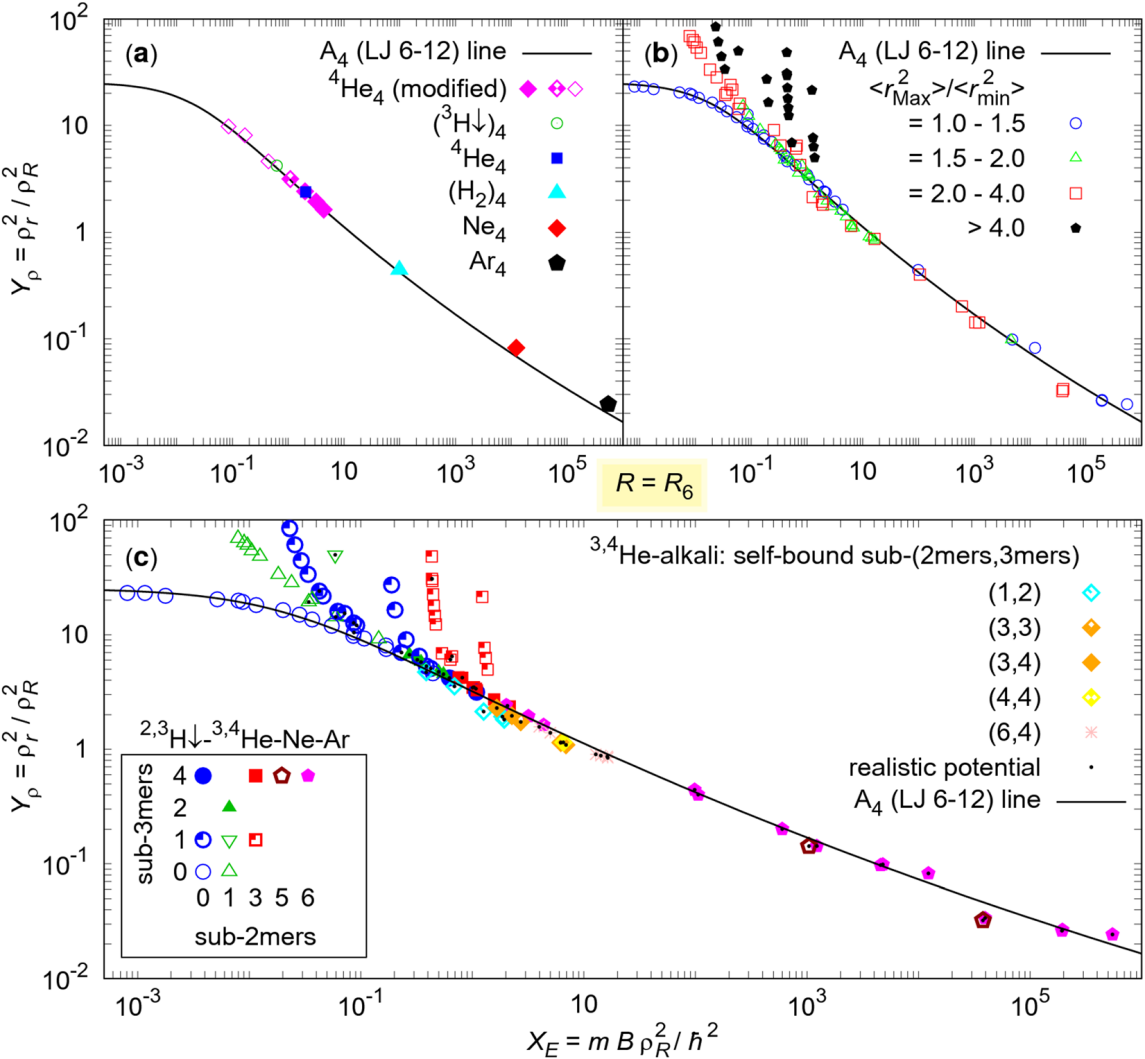
Scaled energies *X*_*E*_ and sizes *Y*_*ρ*_ of various four-body systems for *R* = *R*_6_. The fit obtained in Fig. 1 for homogeneous systems A_4_ is compared with realistic homogeneous systems in (**a**), and with both mixed realistic (additional black dot) and model four-body systems in (**b**) and (**c**). (**a**) Similar symbols as in Fig. 1 were used to distinguish tetramer types. In addition model systems were added for ^4^He_4_. (**b**) Separation of mixed clusters from the homogeneous universal line is analysed. Separation occurs if some pairs of atoms are dominantly extended from the remaining ones. (**c**) Model four-body systems were obtained weakening or strengthening the pair interactions. Different symbols are used to distinguish different types of tetramers with regard to the number of self-bound subsystems of dimers and trimers. Legend for combinations of three helium isotopes and an alkali-atom is given in the top right corner, while types for tetramers consisting of up to three different atoms of spin-polarised hydrogen ^2,3^H↓, ^3,4^He, Ne or Ar are shown in the bottom left corner.

Realistic interaction potentials have more complicated forms than the Lennard-Jones potential. The validity of the *R*_6_ line is tested in Fig. 3a for homogeneous realistic four-body systems. The realistic data were extended by different ^4^He_4_ Lennard-Jones models. Generally, the agreement is very good. Small deviations above the line can occur in the case of a very strong binding, when the potential tail does not have the pure −*C*_6_*r*^−6^ shape, because the higher-order term, −*C*_8_*r*^−8^, is also significant. This is in agreement with the classical result (5), which predicts an increase of the scaled size with *n*. In this context, theoretical results which approximate potentials neglecting higher-order terms, usually predict higher coefficients in Eq. (2) than experimental results.

Mixed four-body systems with original and tuned realistic potentials are compared in Fig. 3c. Constituents and tetramer types are distinguished with different symbols (see the legends). Available published data were taken from helium^20^, helium-alkali^21^, and helium-tritium tetramers^31^. Necessary additional features, like the structure properties 〈*r*^2^〉, and new data for all other clusters included in the Figure were calculated. The energies and scaled values for additional combinations of up to three different isotopes of helium and hydrogen are given in Table 2.

**Table 2:** Ground-state properties.

^2^H	^3^H	^3^He	^4^He	−*E/*mK	*X*_*E*_	*Y*_*ρ*_
1	3			9.9 (1)	0.04	24.1
	3	1		22.5 (7)	0.09	12.1
1			3	135.7 (9)	0.44	30.8
	1	1	2	101.6 (8)	0.35	5.8
	2	1	1	23.5 (9)	0.09	10.5
1	1		2	18 (1)	0.06	50.0

Energy *E* and size, scaled using *R*_6_, i.e., *X*_*E*_ and *Y*_*ρ*_, are reported for tetramers consisting of helium and spin-polarised hydrogen isotopes.

In Fig. 3c one can see that some mixed four-body systems are close to the homogeneous tetramer line, while others show significant deviations from the line. Figure 3b helps to understand the origin of these differences. Complexity grows with different species of particles, namely, particles may have different masses, while the interactions may have different scattering lengths and effective ranges which are not in general correlated with different van der Waals lengths. Tetramers can show many different shapes depending on many factors and sometimes one pair is in a forbidden region, whereas other particles are in an allowed region. If the wave function of a particular sub-dimer heals into much larger distances than the remaining part of the system, it contributes significantly to the size, but, due to the weak binding, very little to the energy. Thus, this system grows in size faster than a homogeneous cluster, producing deviations above the universal line. Mean squares $\langle {r}_{ik}^{2}\rangle$ are convenient for quantifying the spatial extent of any particular pair of particles *i* and *k*. In order to quantify the difference with the homogeneous tetramers, we explored the ratio of the largest and smallest mean square separation of two particles in a particular tetramer. The separation from the homogeneous line is barely visible for the ratio max〈*r*^2^〉/min〈*r*^2^〉 < 1.5, small in the range 〈1.5, 2〉, and significant only for extreme halos in the range 〈2, 4〉. Finally, they are of course very pronounced for max〈*r*^2^〉/min〈*r*^2^〉 > 4. For a particular pair extent ratio, the deviation is larger when the scaled energy *X*_*E*_ is lower.

As shown in Fig. 3c, after separation from the homogeneous line, (0, 1) and (3, 1) tetramer types, which are presented by quarter-full circles and quarter-full squares, respectively, follow two different lines each. However, particular mixed tetramers (*N*_2_, *N*_3_) can go above the universal law following many different lines. The classification of tetramer types (*N*_2_, *N*_3_) was motivated by the suggested^22,27,28^ increase of three-body sizes with *N*_2_, for fixed energies. One could assume ordering *Y*_*ρ*_(*N*_2_ − *n*, *N*_3_) < *Y*_*ρ*_(*N*_2_, *N*_3_) or *Y*_*ρ*_(*N*_2_, *N*_3_ − *n*) < *Y*_*ρ*_(*N*_2_, *N*_3_) in four-body systems. Unfortunately, due to the observed large variability of our results one can identify opposite cases in Fig. 3c. For example, the full square (3, 4) exists below the quarter-full square (3, 1), and the down triangle (1, 1) is between two mentioned lines of quarter-full circles (0, 1). A general statement is only valid for four-body Brunnian system (0, 0); if (0, 0) type exists for given *X*_*E*_, it has the lowest scaled size among other tetramer types.

Reducing the variability, e.g. by fixing the scattering lengths, one can draw additional conclusions. When the system with a bound sub-system approaches the lowest continuum threshold, the size should increase without bound. Let’s consider, for instance, the tetramers A_3_B with the fixed two-body AA scattering lengths, while weakening the AB interactions with the fourth particle B. The more compact A_3_ is, the larger scaled energy *X*_*E*_ is, and therefore system is positioned more to the right in Fig. 3c. Weakening causes that scattering length AB goes to infinity, but particle B keeps moving back and forth between different AB pairs and still feels effectively attractive interaction. This way, B can go at distances much larger than the range of the AB interaction, until further weakening causes that tetramer vanishes at particle + trimer scattering threshold. The order *Y*_*ρ*_(6, 4) < *Y*_*ρ*_(3, 4) < *Y*_*ρ*_(3, 1) is therefore expected and observed in Fig. 3c. Weakening the AB attraction, particle B causes the growth of the wavefunction tail and so it starts preferring to be far away in a classically forbidden region of space. The weaker attraction AB is, the less particle B contributes to the *X*_*E*_, with respect to the amount and the percentage of contribution. Oposite is valid for the scaled size *Y*_*ρ*_. Since *X*_*E*_ decreases negligibly, while *Y*_*ρ*_ simultaneously experiences accelerated increase and diverges, the tetramer A_3_B follows a line which must separate from the universal line. The larger *X*_*E*_ of the separation point is, the steeper the separated line is. This is e.g. observed in quarter-filled squares. Similar features are observed in other complex mixed tetramers.

Additionally, when a system is partially constrained by asymmetrical potential barriers, a mixed system can exhibit also small deviations below the universal line. For instance, an alkali atom in He-alkali tetramers^21^ prevents symmetric configurations: the helium atoms group together in one side of the alkali atom instead of surrounding it, limiting the size of the cluster.

## Discussion

Universal phenomena in few-body systems are mostly discussed in the context of weak binding, but here we extended it from the quantum halo regime all the way to the classical regime. For that purpose, the quantum definition of the van der Waals length *R* = *R*_6_, which is easily calculable and extensible, was also introduced in the scaling of classical systems. For homogeneous tetramers, which feel long-range attraction −*C*_6_*r*^−6^ characteristic for the interaction of neutral atoms, the universal ground-state size-energy law was derived for different scaling lengths *R*. In the case of medium and weakly bound systems, it was shown that the scaled size *Y*_*ρ*_ for *R* = *R*_6_ shows non-linear non-logarithmic shape (3), which was obtained for scaled energies *X*_*E*_ < 10^6^; while the classical result (5), which is linear in log-log scale, can be used for *X*_*E*_ > 10^3^. No significant dependence on short-range interactions indicate the existence of an effective potential barrier which prevents particles from simultaneously getting close together, similarly to the origin of the universal three-body parameter^25^. If the spatially largest configurations of a strongly-bound system significantly experience additional long-range power-law terms −*C*_*n*_*r*^−*n*^ of the potential, the system can slightly deviate from the line, in accordance with the Eq. (5).

The universal size-energy line is also applicable to mixed four-body systems, if their structure is homogeneous-like. This happens when interactions are not highly anisotropic, when no asymmetrical interactions are mediated and all particles have similar probabilities to be far away from the geometric center. Very different potential barriers can limit how particles arrange in the system. When this results in reduced size, like in the case of helium-alkali tetramers, the corresponding systems may appear slightly below the line. If some Jastrow two-body correlation factors^31^ have significantly larger tails than others, deviations above the line might occur, the more easily for weakly bound systems. If the system is not extremely weakly-bound, *X*_*E*_ > 0.1, and the largest mean square pair separation is less than four times the smallest one, the separation from the homogeneous universal line is negligible. By weakening the binding energy of the particles, which already extend significantly in the classically forbidden region, the separation will happen the more abruptly the more compact is the other part of the system, and for that reason it occurs for lower *Y*_*ρ*_.

As the scaled energy goes to the binding threshold, the scaled size of the homogeneous tetramer monotonously rises toward a constant value, which for *R* = *R*_6_ equals 25. The reported size is extrapolated from exact ground-state results and it does not depend on the Lennard-Jones 6–12 potential parameters. In fact, previous variational estimates^12,30^ also predicted finite size. This behavior is opposite to the dimer and trimer cases whose scaled size diverges in both cases^3,12,15^.

The recently developed experimental techniques of Coulomb explosion imaging enable the measurement of structural properties in few-body systems^8,16–19^. Pair distribution functions can be reconstructed from the detected particle positions. Furthermore, it was demonstrated recently^32^ that the potential of diatomic van der Waals systems can be extracted from Coulomb explosion imaging data. Therefore mean square radius for every pair can be calculated and subsequently the sizes *ρ*^2^ and *Y*_*ρ*_. Using the universal law reported in this work, one could recover experimentally usually hardly accessible ground-state energies of weakly bound atomic clusters. Finally, the observed scaling can be used for testing the validity of theoretical models in different fields from ultracold atoms, where interactions can be experimentally tuned, to nuclear physics, where interactions are typically less known than in atomic and molecular physics.

## Methods

The Schrödinger equation written in imaginary time *τ* = *it*/*ℏ*,

$$-\frac{\partial {\rm{\Psi }}({\bf{R}},{\rm{\tau }})}{\partial \tau }=(H-{E}_{{\rm{r}}}){\rm{\Psi }}({\bf{R}},\tau ),$$

was solved at the temperature of 0 K for four-body systems consisting of up to three different components: spin-polarised H and He isotopes, an alkali atom, Ne, Ar, and hydrogen molecules H_2_. Realistic systems were supplemented by different model systems. The positions of atoms were respectively stored in the so-called *walker* ***R*** ≡ (***r***_1_, ***r***_2_, ***r***_3_, ***r***_4_). A stochastic approach was applied by means of the second-order diffusion Monte Carlo method^33^ in which pure estimators^34^ were implemented. Masses and trial wave-functions were taken from our previous works^15,20,21,31^, while the same bias removal approach was applied.

## Data Availability

The data that support the plots within this paper and other findings of this study are available from the corresponding author upon reasonable request.
